# *NOTCH2* variant D1853H is mutated in two non-syndromic premature ovarian insufficiency patients from a Chinese pedigree

**DOI:** 10.1186/s13048-020-00645-4

**Published:** 2020-04-20

**Authors:** Lin Li, Fan Feng, Minying Zhao, Tengyan Li, Wentao Yue, Xu Ma, Binbin Wang, Chenghong Yin

**Affiliations:** 1 Central Laboratory, Beijing Obstetrics and Gynecology Hospital, Capital Medical University, Chaoyang, Beijing, 100026 China; 2 Department of Basic Medical Sciences, School of Medicine, Tsinghua University, Haidian, Beijing, 100084 China; 3 Department of Reproductive Medicine, the First Hospital of Shijiazhuang, 36 Fanxi Road, Shijiazhuang, 050011 Hebei China; 4 Center for Genetics, National Research Institute for Family Planning, 12, Dahuisi Road, Haidian, Beijing, 100081 China

**Keywords:** Premature ovarian insufficiency, NOTCH2, Whole-exome sequencing, RNA sequencing, Gene ontology, Collagen degradation

## Abstract

**Background:**

Premature ovarian insufficiency (POI) is a severe disorder of female infertility, characterized by 4–6 months of amenorrhea before the age of 40 years, with elevated follicle stimulating hormone (FSH) levels (> 25 IU/L). Although several genes have been reported to contribute to the genetic basis of POI, the molecular mechanism of POI remains unclear.

**Methods:**

Whole-exome sequencing (WES) was performed. Sanger sequencing was carried out to validate the variant in the proband and her mother. In silico algorithms were used to analyze the mutational effect of the variant. Protein 3D structural modeling was used for predicting mutated protein structures. Vector construction and plasmids transfection were performed, and subsequently RNA-sequencing (RNA-seq) was carried out in each group to dissect the differentially expressed genes in wild-type (WT) and D1853H NOTCH2 mutant expressing groups. Gene Ontology analysis was also used to analyze the enriched biological processes or pathways among the differentially expressed genes.

**Results:**

We report two non-syndromic POI patients from a Chinese pedigree. The FSH level of the proband (the daughter) was 46 IU/L at the age of 22. Her menarche was at the age of 12, but she was amenorrhea at the age of 20. By WES, a rare heterozygous variant (c.5557G > C;p.D1853H) in the *NOTCH2* gene was identified. In silico analysis suggested that p.D1853H was a pathogenic allele. Protein 3D structural modeling suggested that D1853H may enhance or weaken the electrostatic surface potential. By molecular analysis, we found that cells expressing the D1853H NOTCH2 mutant had similar effect in activating the NOTCH signaling pathway downstream target genes. However, 106 protein-coding genes were differentially expressed between D1853H expressing cells and WT NOTCH2 expressing cells, and these genes were enriched for collagen degradation, NCAM1 interactions and HDACs deacetylate histones, revealing a unknown underlying mechanism of the pathology that leads to POI.

**Conclusions:**

We conclude that the rare heterozygous variant in *NOTCH2* may be associated with POI. This finding provides researchers and clinicians with a better understanding of the etiology, molecular mechanism and genetic consulting of POI.

## Background

Premature ovarian insufficiency (POI), also known as premature ovarian failure or primary ovarian insufficiency, is a severe disorder causing human female infertility. POI is defined as premature ovarian defect before the age of 40 years, and is characterized by premature depletion of ovarian follicles and 4–6 months of amenorrhea, with elevated FSH levels (> 25 IU/L) [1]. POI is genetically heterogeneous, and its molecular mechanism is still unclear. Several genes have been reported to contribute to the genetic basis of POI, including *FMR1*, *FSHR*, *NOBOX*, *BMP15*, *NR5A1*, *POLG*, *FIGLA*, and *FOXL2* [2–9]. In recent years, WES technology has been used to identify the genetic causes of familial POI and these analyses have identified several novel genes associated with POI, such as *STAG3*, *HFM1*, *MCM8*, *MCM9*, *BRCA2*, *FANCA*, *TP63*, *POLR3H*, *MEIOB*, *DMC1*, *KHDRBS1*, *EIF4ENIF1* and *BNC1* [10–24]. These findings indicated that WES is a powerful tool for dissecting the genetic alterations in familial POI.

Here, we report on two patients with POI in a Chinese pedigree. By WES, we found that the patient with POI (the proband) was heterozygous for the missense variant c.5557G > C;p.D1853H of the *NOTCH2* gene that she inherited from her mother. Further functional analysis revealed that p.D1853H may not affect the NOTCH signaling pathway, but exerted a deleterious effect on the biological processes of collagen degradation, NCAM1 interactions and HDACs deacetylate histones through an unknown pathway, demonstrating that the rare heterozygous variant p.D1853H in *NOTCH2* may be associated with POI.

## Methods

### Patients

Two non-syndromic POI patients from a Chinese pedigree were recruited from The First Hospital of Shijiazhuang. POI was diagnosed if the patients had amenorrhea for at least 6 months before the age of 40 and two consecutive FSH measurements > 25 IU/L taken 2 months apart [1]. The two POI patients did not show any of the following: karyotypic abnormality, autoimmune disorder, history of radiotherapy and chemotherapy, or pelvic surgery. The hormones levels of the proband (the daughter) were as follows: FSH, 46 IU/L; luteinizing hormone (LH), 20.48 IU/L; testosterone (T), 2.04 nmol/L; estradiol (E2), 82.96 pmol/L; prolactin (PRL), 151.4 μIU/mL. The uterus and the ovaries of the proband were found to be atrophic by transvaginal color Doppler ultrasound examination. Therefore, the proband was diagnosed as POI, and the proband was 20 years old at the time of diagnosis. Her menarche was at the age of 12, but she had amenorrhea after the age of 20. Her height, weight and body mass index were 1.66 m, 52 kg and 18.87 kg/m^2^, respectively. Medical examination revealed no Hajdu-Cheney Syndrome (characterized as acro-osteolysis, generalized osteoporosis, craniofacial anomalies and renal cysts) (Supplemental Fig. 1A and B) or Alagille syndrome (characterized as cholestatic liver disease, cardiac disease, ocular abnormalities, skeletal abnormalities and characteristic facial features). The proband has been treated with estrogen and progesterone artificial cycle therapy since amenorrhea since June 2013. The drug is Femoston (estradiol tablets / estradiol and dydrogesterone tablets in a compound package, Solvay Pharmaceuticals B.V., 28 tablets/box, the first 14 tablets are red film-coated tablets containing 2 mg of 17-β-estradiol, and the latter 14 tablets are yellow film-coated tablets containing 17-β-estradiol 2 mg and dydrogesterone 10 mg.). 28 days is a cycle. The proband takes it for 3 consecutive years. After that, the patient suspends the medicine for one year. During the period of the withdrawal, the patient’s menstrual flow naturally occurs twice. The patient has been taking Femoston continuously since October 2018. During the period of medication, her menstruation can come regularly. There are no adverse reactions to the medication.

The proband’s mother was 47-year-old. Her menarche was at the age of 13, but she had amenorrhea at the age of 35. The uterus and the ovaries of the mother were also found to be atrophic by transvaginal color Doppler ultrasound examination. POI was diagnosed when the mother was 36-year-old. Medical examination also revealed no Hajdu-Cheney Syndrome (Supplemental Fig. 1c and d) or Alagille syndrome in the mother. This study was approved by the Ethics Committee of the First Hospital of Shijiazhuang. Written informed consent was obtained from each participant. 5 ml of peripheral blood was collected.

### WES and sanger sequencing validation

WES was carried out using TruSeq Exome Enrichment Kit on the Illumina Hiseq 4000 platform as previously described [18]. We hypothesized that heterozygous variants were in accord with the POI mode of inheritance in this pedigree. Therefore, variants fulfilling the following criteria were retained for subsequent analyses: (i) missense, nonsense, frame-shift, or splice site variants; (ii) absent or rare (frequency < 0.1%) in dbSNP, 1000 Genomes, Exome Aggregation Consortium (ExAC) and in-house databases. Sanger sequencing was used to validate the variant in both the proband and her mother.

### Vector construction

Because human NOTCH2 is resistant to ligand-independent activation [25], we used the 937 amino-acids of NOTCH2 intracellular domain sequence (NICD) (Ala^1535^-Ala^2471^) [26] to study the functional alteration of NOTCH2, as the D1853H variant is located in the NICD region. The human NOTCH2 NICD coding sequence was amplified by PCR from a commercial plasmid (YouBio, G152986, China). The human NOTCH2 NICD coding sequence was subcloned into the pHS-BVC-LW450 vector, which contained a 3× flag and internal ribosome entry site (IRES) - enhanced green fluorescent protein (EGFP) sequences (Supplemental Fig. 2A). We renamed this construct as NOTCH2-WT. A site-directed mutagenesis approach (Hieff Mut™ Site-Directed Mutagenesis Kit, Cat. No 11003ES10, China) was used to introduce the c.5557G > C (p.D1853H) variant into this plasmid, and this construct was named as NOTCH2-D1853H. The inserted sequences were further confirmed by Sanger sequencing.

### Cell culture and plasmid transfection

The procedures of culture, passage and plasmid transfection of 293FT cells were performed as previously described [2, 27].

### RNA-sequencing and data analysis

RNA-sequencing was performed as previously described [28] by Annoroad Gene Technology Co., Ltd. (Yiwu, China). Genes with significant *p*-values (*p* < 0.05) were considered differentially expressed. Gene Ontology (GO) analysis was carried out using the gene annotation and analysis resource Metascape (http://metascape.org/gp/index.html).

### Protein structural modeling

I-TASSER (Iterative Threading ASSEmbly Refinement) is a program to predict protein structure and function [29, 30]. The program first tries to find out template proteins of similar folds from the Protein Data Bank (PDB). Next, the continuous fragments adopted from the PDB templates are reassembled into full-length models. The low free-energy states are identified by SPICKER through clustering the simulation decoys. In the third step, the fragment assembly simulation is performed again. The NOTCH1–4 alignment was performed using ClustalW and ESPript 3.0.

## Results

### WES analysis of the patients with POI

**Fig. 1:**
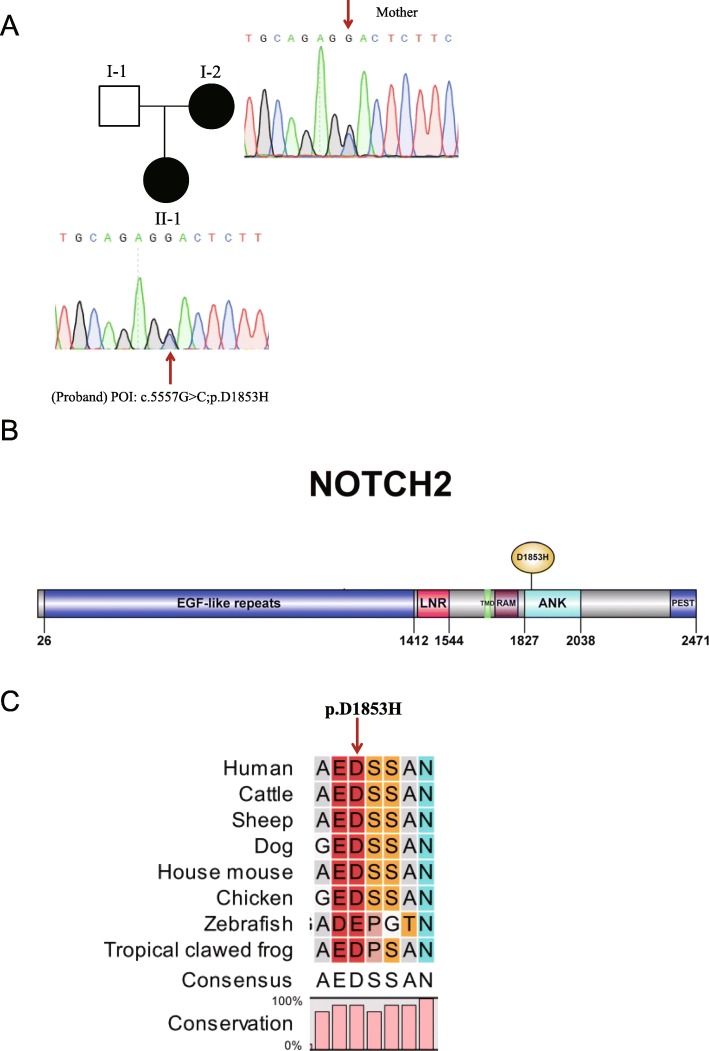
*NOTCH2* variant in POI patients. (A) Two POI patients in a Chinese pedigree. Each of the affected family members (black circle) carried a heterozygous *NOTCH2* mutation. The red arrows point to the mutation site. (B) Domains and mutation site in the NOTCH2 protein. The full-length protein contains 2471 amino acids (aa). EGF-like repeats, aa 26–1412 (blue box); ANK domain, aa 1827–2038 (light blue box). The D1853H variant located in the ANK domain, respectively. (C) Alignment of NOTCH2 proteins from different species. The D1853 site of human NOTCH2 is highly conserved in the aligned sequences

Whole-exome sequencing was performed in samples from the POI proband and her mother (with POI). Pedigree analysis suggested a dominant mode of inheritance associated with POI (Fig. 1a). After filtering out polymorphisms with allele frequency greater than 0.1% in the dbSNP, 1000 Genomes, ExAC and our inhouse databases, we compiled a list of genes harboring heterozygous variants in both the proband and her mother (Supplemental Table 1). Among these 87 genes (Supplementary Table 1), considering changes in conserved amino acids and published phenotypes of knock-out mice, we found that knock out of *Notch2* or *Nr6a1* resulted in defective follicle development in mice [31, 32]. Therefore, we hypothesized that the heterozygous variant in the *NOTCH2* or *NR6A1* gene was associated with POI. However, the same variant in the *NR6A1* gene was found in a screen of 50 healthy control women. Therefore, the heterozygous variant in the *NR6A1* gene was excluded. We performed Sanger sequencing and confirmed that the heterozygous mutation in *NOTCH2* (c.5557G > C; p.D1853H) was present in the proband and her mother (Fig. 1a).

### In silico analysis of the sequence variant

In silico analysis predicted that the *NOTCH2* p.D1853H (abbreviated as D1853H) variant is likely to be a disease-associated mutation by four online prediction tools (Table 1). The allele frequency of c.5557G > C in East Asian population in the ExAC database were 0.0002326, indicating the rarity of the sequence variant. D1853 is located in the ankyrin (ANK) domain in the NOTCH2 protein (Fig. 1b). The aspartic acid located at position 1853 in human NOTCH2 is highly conserved among different species from human to tropical clawed frog (Fig. 1c), indicating its functional importance.

**Table 1:** In silico analysis of the *NOTCH2* variant

Mutation	Amino acid change	Polyphen-2^a^	SIFT^b^	Mutation Taster^c^	SNPs&GO^d^	ExAC (total)^e^	ExAC (East Asian)^f^
c.5557G > C	p.D1853H	Probably damaging (0.996)	Damaging (0.02)	Disease causing (0.9999)	Disease (0.831)	1.666E-05	0.0002326

^a^Polyphen-2 (http://genetics.bwh.harvard.edu/pph2/). Prediction Scores range from 0 to 1 with high scores indicating probably or possibly damaging

^b^SIFT, i.e., Sorting Intolerant From Tolerant (http://sift.jcvi.org/). Scores vary between 0 and 1. Variants with scores close or equal to 0 are predicted to be damaging

^c^Mutation Taster (http://www.mutationtaster.org/). The probability value is the probability of the prediction, i.e., a value close to 1 indicates a high ‘security’ of the prediction

^d^SNPs&GO (http://snps.biofold.org/snps-and-go/). Disease probability (if > 0.5 mutation is predicted Disease)

^e^Frequency of variations in total of ExAC database

^f^Frequency of variations in East Asian population of ExAC database

### Protein structural modeling analysis of D1853H

**Fig. 2:**
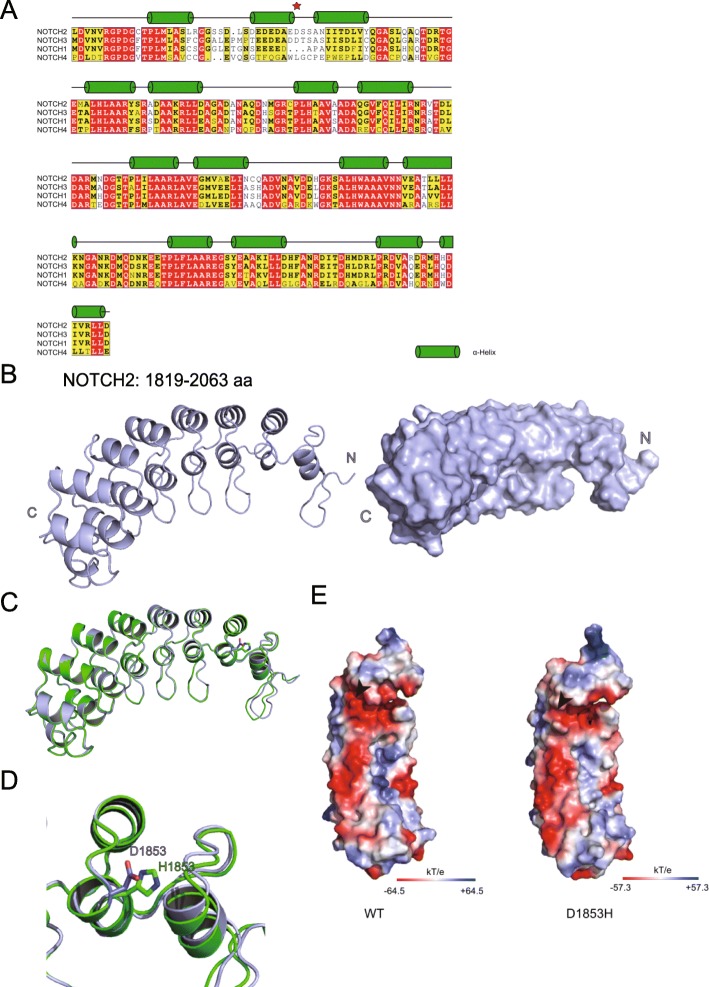
3D structural modeling of NOTCH-WT and NOTCH-D1835H. (A) Sequence alignment and secondary structure prediction of NOTCH ANK domains. Multiple sequence alignment of the ANK domain of human NOTCH1–4. The secondary structure assignment of human NOTCH2 is shown above the alignment. Helices are labeled with green cylinder. Red star represents D1853. Residues in red boxes are absolutely conserved; those in yellow boxes are conserved in several of the homologues. (B) 3D structure of NOTCH2 ANK domain (1819–2063 amino acid (aa)). The structure is shown as cartoon (left) and surface (right). (C) 3D structure alignment of NOTCH2-WT (blue) and -D1853H (green) ANK domain. Alignment is shown as cartoon. (D) Close view of 1853 site in the ANK domain. D1853 side chain is shown as blue and H1853 side chain is shown as green. (E) Electrostatic potential of the ANK domain of NOTCH2-WT and -D1853H. WT (left) electrostatic potential is expressed as a spectrum ranging from − 64.5 kT/e (red) to + 64.5 kT/e (blue); D1853H (right) electrostatic potential is expressed as a spectrum ranging from − 57.3 kT/e (red) to + 57.3 kT/e (blue)

There are four mammalian NOTCH homologues (NOTCH 1–4). Their ANK domains share significant sequence identity. By sequence alignments, the conserved amino acids located at important sites were identified (Fig. 2a). ANK domains are generally responsible for mediating protein-protein interactions, which have been shown to interact with several proteins, including p300 and PCAF [33, 34]. This information indicated that the ANK domain may recruit other co-activators for gene transcription.

ANK repeats have been found in many proteins and they have a conserved secondary and tertiary structure, although there is sequence variability among them. Based on the I-TASSER program, we predicted the ANK domain secondary and tertiary structures. Firstly we analyzed the predicted secondary structure of NOTCH2 ANK domain. We found that there were seven pairs of α-helices in the ANK region and The pairs 2–7 contained conserved amino acids compared with other orthologs (Fig. 2a). The first pair of helices was not conserved as it was variable across the different alleles. This result may indicate that this region is involved in different functions. Further, more importantly, the mutation D1853H is just located in this region.

Next, we predicted the higher order structure of NOTCH2 ANK domain. According to the secondary structure prediction result, there were seven pairs of helices in ANK domain and the NOTCH2 ANK domain was slightly curved, a feature that appeared to be the result of different helix lengths and inter-repeat interactions between the helices (Fig. 2b). By analyzing the protein structure, we discovered that a low conservation amino acid region is located at the first pair of α-helices. In comparison with WT, the structure of the D1853H mutant protein did not show big differences (Fig. 2c), only the side chain was changed (Fig. 2d). Next, we compared the WT protein and mutant protein surface electronic charge (Fig. 2e). We found that there was a slight change between WT and mutant protein. The D1853 site is located at the first pair of helices, and the 3D structure showed that this region may form a “pocket” on the protein surface, which indicated that the N terminal may be an important protein-protein interaction region and this pocket may play a key role in NOTCH2 binding to other proteins. The fact that D1853H is located in this region and the amino acid change may enhance or weaken the electrostatic surface potential, suggesting that it may be a significant regulator of NOTCH2.

### Molecular analysis of the variant’s effect

NOTCH receptor signaling plays a key role in animal development, and is involved in cell fate decisions. NOTCH receptor consists of a large extracellular domain, containing epidermal growth factor (EGF) and Lin/Notch Repeats (LNR), and an NICD, which includes a region comprising several ANK repeats (Fig. 1b). Previous reports have reported that mutations in ANK repeats affect NOTCH signaling. As the D1853H variant is located in the ANK repeats, we hypothesized that it affects the NOTCH signaling pathway. Therefore, we comprehensively examined the effect of D1853H on gene transcription. We subcloned the NICD WT sequence (for details, please see the Material and Methods section) in an expression vector (Supplementary Fig. 2A), and we also introduced the c.5557G > C (p.D1853H) mutation into the same vector, and obtained the NOTCH2-D1853H plasmid (Supplementary Fig. 2B).

**Fig. 3:**
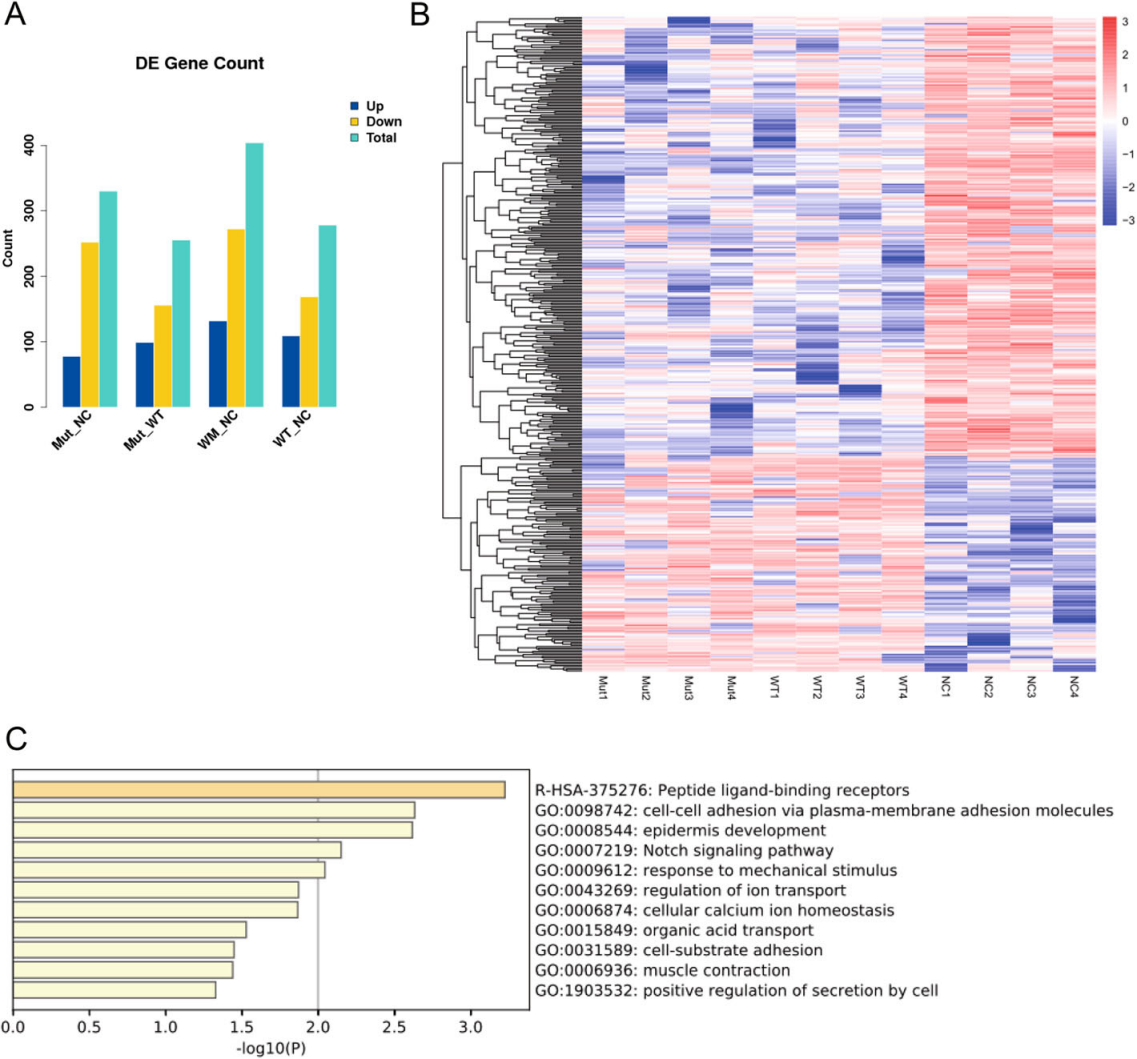
RNA-sequencing analysis of wild-type (WT), D1853H NOTCH2 (Mut), and negative control (NC) cells. (A) The overall differentially expressed (DE) gene count in different comparison groups. WT and Mut are collectively designated as WM. (B) Heatmap data from the RNA-sequencing analysis. Overall, 132 transcripts, including 51 protein-coding genes, were significantly increased in WM group compared with the NC group. The expression of 272 transcripts decreased significantly. Red and blue indicate expression at relatively high and low levels, respectively. The experiment was conducted four times. (C) Gene Ontology (GO) analysis of the 51 protein-coding genes described the biological processes of the increased expression genes in WM cells compared with those in NC cells

Then, the same amount of the NOTCH2-WT plasmid, NOTCH2-D1853H plasmid, and the empty vector (pHS-BVC-LW450) were transfected in 293FT cells. After two days, the cells were collected, RNA was extracted and subsequently RNA sequencing was carried out for each group (NOTCH2-WT expressing cells were abbreviated as WT, NOTCH2-D1853H expressing cells were abbreviated as Mut, empty vector cells were abbreviated as NC). For each group, four independent replicates were used. Following bioinformatic analysis of the RNA sequencing data, we found that 132 transcripts, including 51 protein-coding genes, were significantly upregulated in WT and Mut (wholly known as WM) groups compared with the NC group (Fig. 3a and b, Supplementary Table 3). The expression of 272 transcripts (*p* < 0.05) was significantly downregulated in WM groups (Fig. 3a and b, Supplementary Table 3). Gene Ontology (GO) analysis of the 51 protein-coding genes (*p* < 0.05) showed that NOTCH signaling pathway was significantly enriched (Fig. 3c), indicating that both the NOTCH2-WT and NOTCH2-D1853H could activate the NOTCH signaling pathway. Specifically, we first examined the expression of the *NOTCH2* gene, and found that there was no significant difference in the expression levels between WT and Mut in transfected cells (Supplementary Fig. 3A), which indicated that the transfection and expression levels of plasmids in WT and Mut groups were similar. Furthermore, we found that both WT and Mut groups could activate the expression of *HES5* and *HES1*, two downstream target genes of NOTCH signaling (Supplementary Fig. 3A). Therefore, we concluded that the D1853H variant did not significantly affect the transcriptional activation of NOTCH signaling.

**Fig. 4:**
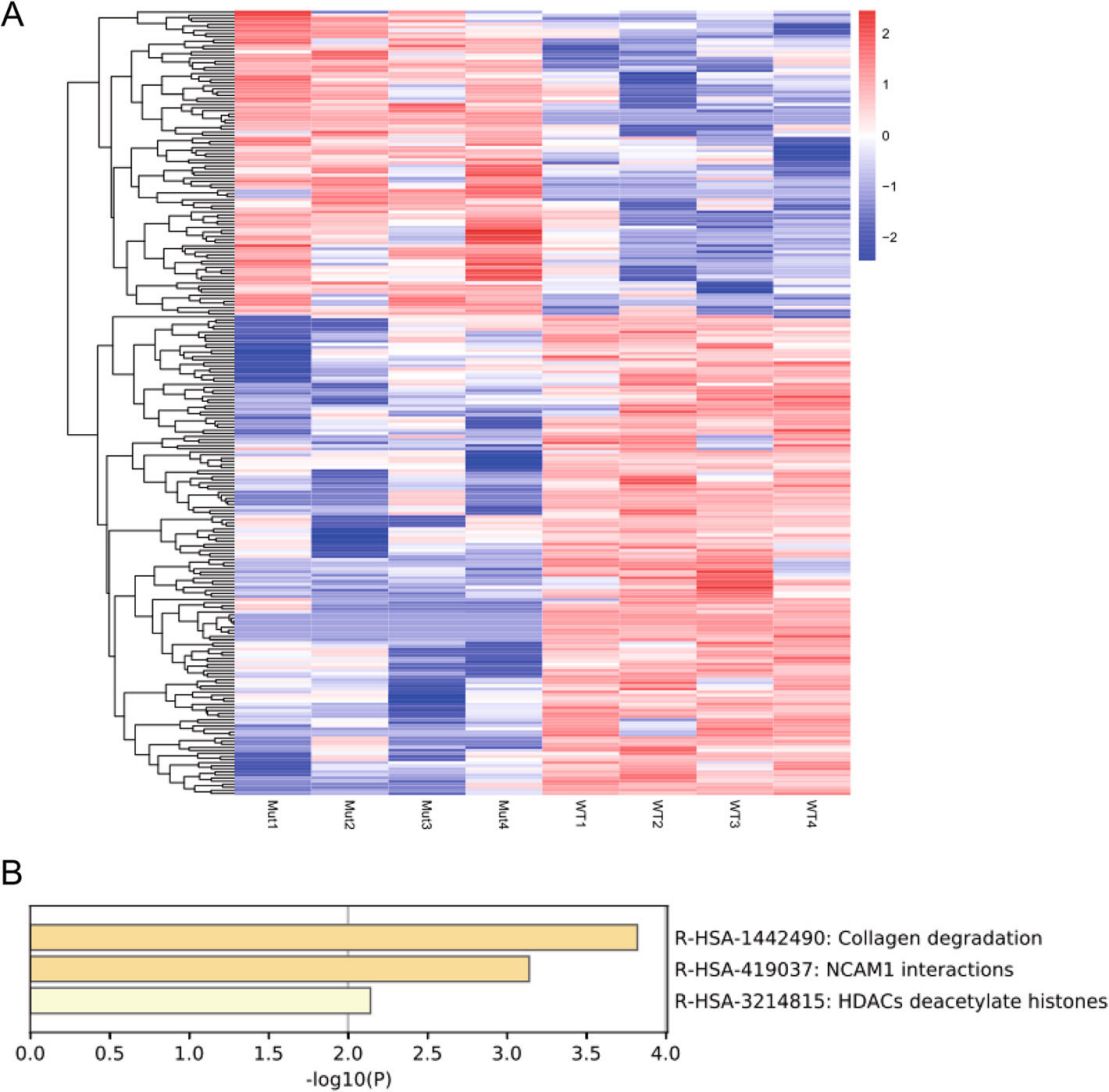
RNA-sequencing analysis of WT and Mut cells. (A) Heatmap data from the RNA-sequencing analysis of WT and Mut cells. The expression of 255 transcripts (including 106 protein-coding genes) was significantly different between the WT and Mut groups. Among them, 54 genes were downregulated and 52 genes were upregulated in the Mut group. (B) GO analysis of the 106 protein-coding genes

We then focused on the differences in gene expression between WT and Mut groups. The expression of 255 transcripts (including 106 protein-coding genes, *p* < 0.05) was found to be significantly different between the two groups (Fig. 3a and 4a, Supplementary Table 4). Among them, 54 genes (*p* < 0.05) were downregulated in the Mut group and 52 genes (*p* < 0.05) were upregulated in the Mut group. GO analysis of the 106 genes indicated that genes related to collagen degradation, NCAM1 interactions and HDACs deacetylate histones (*p* < 0.05) were the most abundant among these differentially expressed genes (Fig. 4b). Therefore, we examined the expression levels of genes related to collagen degradation and germ cell development. The collagen-related genes *MMP13* and *MMP10* were significantly downregulated in the Mut group (Supplementary Fig. 3B), while genes related to germ cell development, such as *GNRH1*, *BOLL* and *NGF*, were also significantly downregulated in the Mut group (Supplementary Fig. 3B). Therefore, the D1853H variant may induce a expression profile change not by canonical NOTCH signaling, but by an unknown mechanism.

## Discussion

The Notch signaling pathway controls many cell fate decision events, and regulatory abnormalities in this pathway may cause many developmental diseases. Notch receptors are single-pass transmembrane proteins and play important roles in the development of many organs, such as kidney, bone and ovaries. *Notch2*, one of the four Notch genes, is abundantly expressed in mouse ovarian granulosa cells [35]. The expression level of *Notch2* and Notch target genes *Hes1* and *Hey2* are increasing from E13.5 to PND0 [31]. *Notch2* conditional knock-out mice are characterized by abnormal follicles with enlarged oocytes without granulosa cell growth, similar to the follicles that undergo premature activation [31]. The number of primordial and antral follicles was reduced at PND19 and PND22.5 in *Notch2* conditional knock-out mice, respectively [31]. Furthermore, apoptosis of granulosa cells was increased [31], explaining the aberrant follicles. A recent study used growth hormone (rmGH) treatment of premature ovarian failure mice and found that plasma estradiol levels increased and plasma follicle stimulating hormone levels decreased via stimulating Notch signaling pathway [36]. The role of NOTCH2 in follicle development and granulosa cell differentiation suggested a link with POI pathogenesis. In support, Patino et al., have reported an association between the *NOTCH2* mutations and POI [26]. They found that p.Ser1804Leu, p.Ala2316Val, p.Pro2359Ala *NOTCH2* mutations can affect the protein’s transcriptional activity [26].

Here, we report a heterozygous variant p.D1853H in the *NOTCH2* gene in two patients with POI in a Chinese pedigree. D1853 is highly conserved and is located in the ANK domain of the NOTCH2 protein. The ANK domain is responsible for RBP-J binding, co-repressor displacement and necessary for transactivation [37], therefore, the D1853H variant was postulated to affect the transcription of target genes, such as *HES1* and *HES5*. However, by RNA-sequencing analysis of the WT and D1853H (Mut) expressing cells, we found that both WT and D1853H could activate the downstream target genes. Furthermore, there was no significant difference in the degree of activation between the WT and D1853H groups. D1853H variant might induce a mild functional change of NOTCH2 not by canonical NOTCH signaling, but by an unknown mechanism. The collagen-related genes *MMP13* and *MMP10* were significantly downregulated in the D1853H group. Matrix metalloproteinases (MMPs) play important roles in extracellular matrix degradation and cell remodeling [38]. MMP13, a type of collagenase that degrades fibrillary collagens, was increased in bovine preovulatory follicles following gonadotropin surge [39], and has been suggested to be involved in the breakdown of the follicle wall [40]. So, downregulation of the collagen-related genes might be associated with the breakdown of follicle during the preovulatory stage. The nerve growth factor *(NGF)* gene was also reduced in the D1853H group. NGF is required for the growth of primordial ovarian follicles, and *NGF* knock-out mice displayed a delay in follicular growth due to the loss of a proliferative signal [41]. NGF and its receptors are also present in human ovaries [42]. The biological process related to NCAM1 interactions were enriched in the 106 differentially expressed genes. NCAM1 was expressed mainly in the granulosa cells of growing preantral and antral follicles and in corpora lutea [43], and has been suggested to be involved in folliculogenesis and the formation of the corpus luteum in the human [44]. Further protein structure analysis showed that the D1853 region appears to form a “pocket” on the protein surface, which indicated that the N terminal of NICD may be an important protein-protein interaction region. Therefore, we hypothesized that D1853H might affect the protein-protein interactions, and thus other unknown pathways.

Previous human genetic studies on the *NOTCH2* gene have revealed that heterozygous truncating mutations in exon 34 cause Hajdu-Cheney syndrome, which is characterized by progressive focal bone destruction, including acro-osteolysis and generalized osteoporosis [45]. In 2012, a Japanese group reported that one girl with Hajdu-Cheney syndrome developed premature ovarian failure [46], indicating that alteration in the *NOTCH2* gene may be also associated with premature ovarian failure. In our report, both the POI patients, proband and her mother, did not develop the Hajdu-Cheney syndrome, probably because the D1853H mutation does not induce a severe transcriptional defect as the truncating mutations in exon 34. Therefore, different mutations in the same gene would lead to different disease phenotypes.

## Conclusions

Taken together, our study demonstrated that the heterozygous variant p.D1853H of the *NOTCH2* gene may be associated with POI, not by affecting the Notch signaling transduction and downstream target gene activation, but by an unknown mechanism. This finding provides researchers and clinicians with a better understanding of the etiology, genetics and molecular mechanisms of POI.

## Supplementary information

**Additional file 1 Fig. S1.** The two POI patients are not syndromic POI and do not have acro-osteolysis. A. The two hands of the daughter are normal. B. The foot of the daughter is normal. C. The two hands of the mother are normal. D. The foot of the mother is normal.

**Additional file 2 Fig. S2.** Construction of plasmids containing the WT or mutant NOTCH2 intracellular domain (ICD). (A) the pHS-BVC-LW450 vector map. The vector contained a 3× flag and internal ribosome entry site (IRES) - enhanced green fluorescent protein (EGFP) sequences. (B) Sanger sequencing validated the WT and 5557C (mut) plasmid sequences.

**Additional file 3 Fig. S3.** Gene expression levels by RNA-seq analysis. (A) NOTCH signaling is activated in WT and 5557C (mut) groups. *NOTCH2* was expressed at approximately 60 folds in WT and 5557C (mut) groups compared with the empty vector transfected group (Ctrl). Expression of *HES5* and *HES1* was high in WT and 5557C groups. (B) Different genes’ expressions between WT and 5557C groups.

**Additional file 4 Table S1.** Heterozygous sequence variants found in the two patients

**Additional file 5 Table S2.** DNA Primers used in this study.

**Additional file 6 Table S3.** 404 differentially expressed transcripts bewteen the WM (WT plus Mut groups) and control groups

**Additional file 7 Table S4.** 255 differentially expressed transcripts bewteen the Mut and WT groups

## Data Availability

The datasets used and/or analyzed during the current study are available from the corresponding author upon reasonable request.
