# Supplementary figures and images for: NOTCH2 variant D1853H is mutated in two non-syndromic premature ovarian insufficiency patients from a Chinese pedigree
Source: J Ovarian Res. 2020 Apr 20;13:41. doi: 10.1186/s13048-020-00645-4 (PMC7171760; doi:10.1186/s13048-020-00645-4)

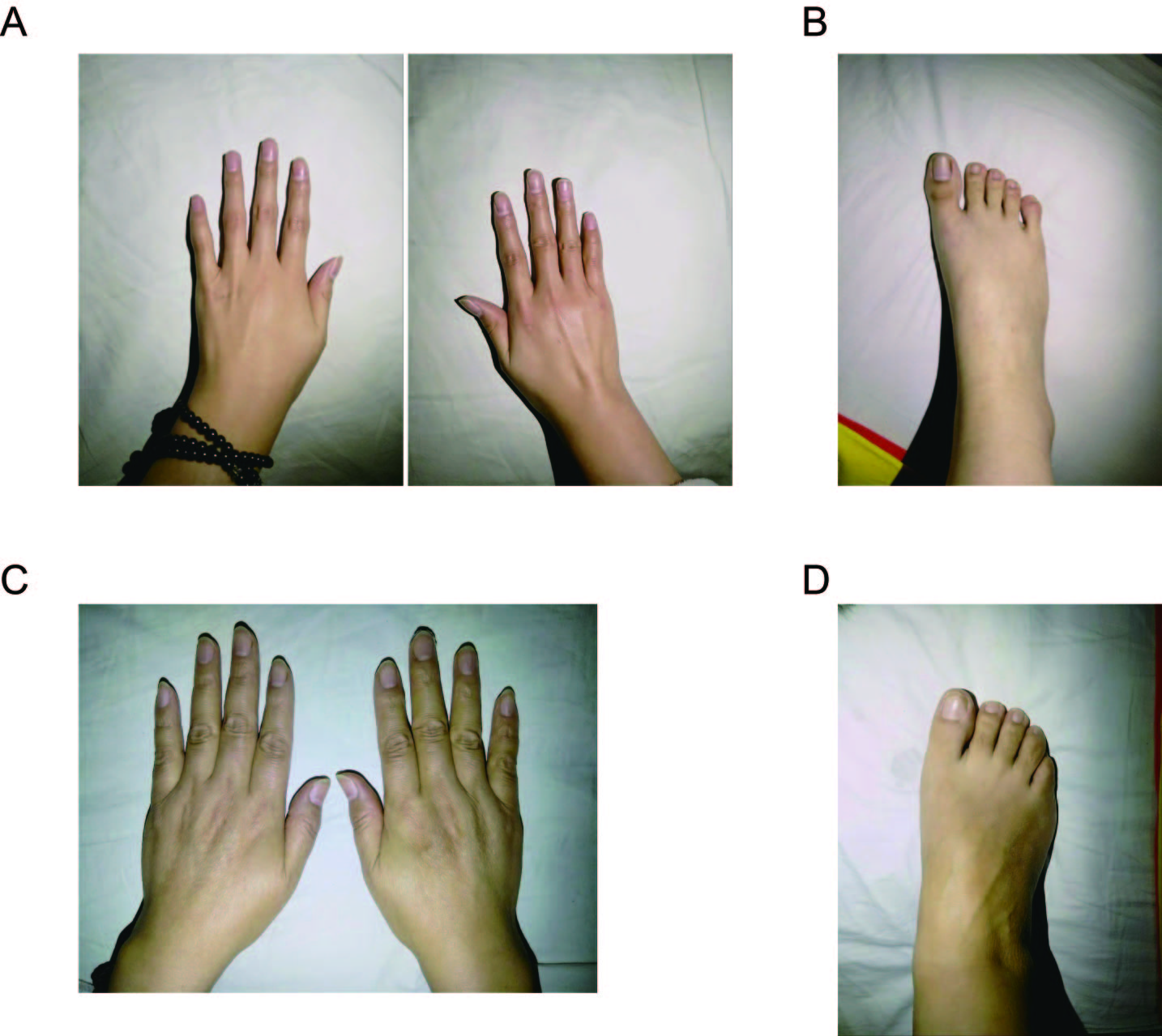

Supplement: Supplementary file 1 — Additional file 1 Fig. S1. The two POI patients are not syndromic POI and do not have acro-osteolysis. A. The two hands of the daughter are normal. B. The foot of the daughter is normal. C. The two hands of the mother are normal. D. The foot of the mother is normal. [file 13048_2020_645_MOESM1_ESM.jpg]

A

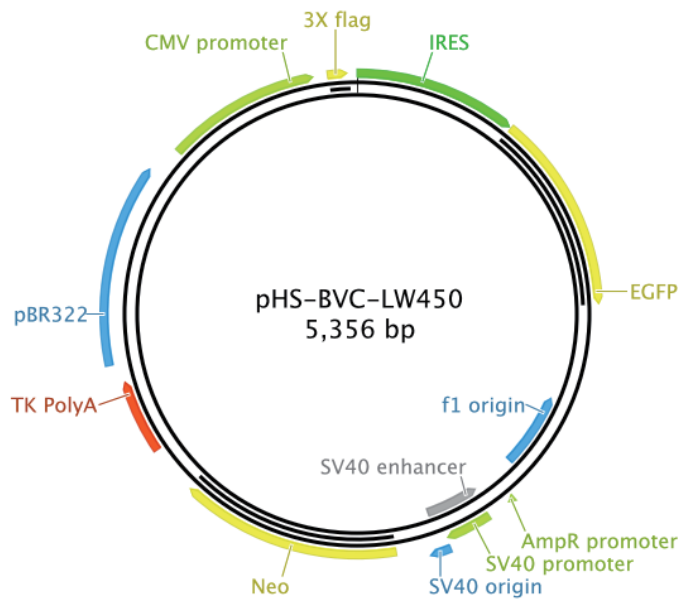

B

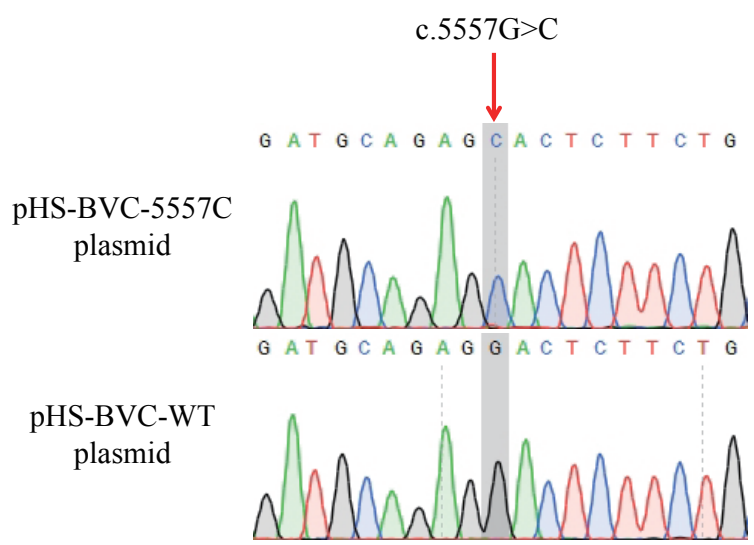

Supplement: Supplementary file 2 — Additional file 2 Fig. S2. Construction of plasmids containing the WT or mutant NOTCH2 intracellular domain (ICD). (A) the pHS-BVC-LW450 vector map. The vector contained a 3× flag and internal ribosome entry site (IRES) - enhanced green fluorescent protein (EGFP) sequences. (B) Sanger sequencing validated the WT and 5557C (mut) plasmid sequences. [file 13048_2020_645_MOESM2_ESM.pdf]

A

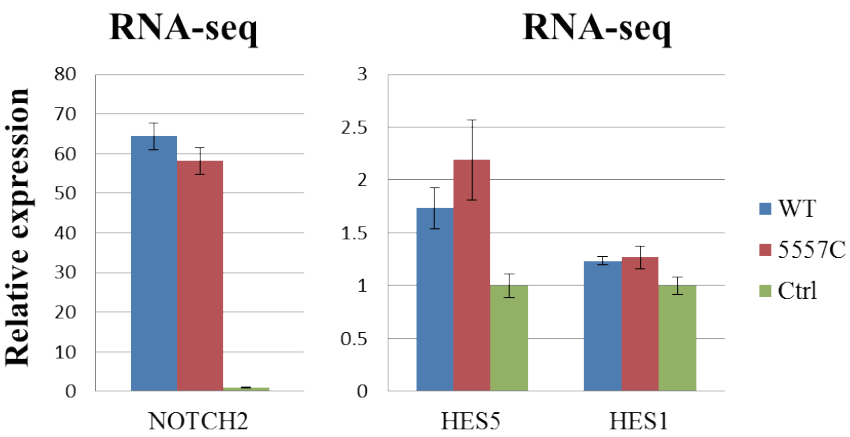

B

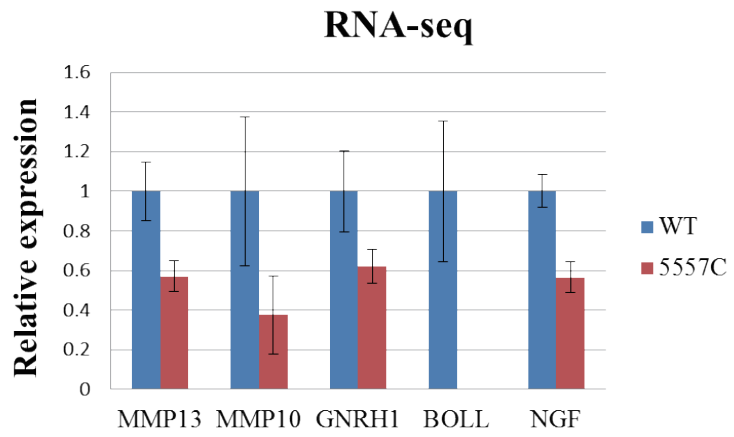

Supplement: Supplementary file 3 — Additional file 3 Fig. S3. Gene expression levels by RNA-seq analysis. (A) NOTCH signaling is activated in WT and 5557C (mut) groups. NOTCH2 was expressed at approximately 60 folds in WT and 5557C (mut) groups compared with the empty vector transfected group (Ctrl). Expression of HES5 and HES1 was high in WT and 5557C groups. (B) Different genes’ expressions between WT and 5557C groups. [file 13048_2020_645_MOESM3_ESM.pdf]
