# Supplementary material for: NOTCH2 variant D1853H is mutated in two non-syndromic premature ovarian insufficiency patients from a Chinese pedigree
Source: J Ovarian Res. 2020 Apr 20;13:41. doi: 10.1186/s13048-020-00645-4 (PMC7171760; doi:10.1186/s13048-020-00645-4)
Supplement: Supplementary file 5 — Additional file 5 Table S2. DNA Primers used in this study. [file 13048_2020_645_MOESM5_ESM.docx]

**Supplementary Table 2.** DNA Primers used in this study.

| **Gene** | **Forward Primer** | **Reversed Primer** | **Experiment** |
| --- | --- | --- | --- |
| *NOTCH2-G5557* | AAGGAATGTAGCCACAAA | TCTTTACCTGGAAGACACC | Mutational analysis in patients |
| *NOTCH2-5557C* | AGGTGGATGTGTTAGATGTG |  | Vector Sanger sequencing validation |
